# Predicting sulfur solubility in hydrogen sulfide, carbon dioxide, and methane with an improved thermodynamic model

**DOI:** 10.1039/c8ra01744a

**Published:** 2018-04-30

**Authors:** Changjun Li, Gang Liu, Yang Peng

**Affiliations:** School of Petroleum Engineering, Southwest Petroleum University Chengdu 610500 China sci-tech@hotmail.com +86 132 2817 0900; Gas Management Office, PetroChina Southwest Oil & Gas Field Company Chengdu 610215 China

## Abstract

During development of high sulfur-content natural gas fields, gaseous sulfur is likely to precipitate and deposit in the reservoir and transmission pipelines owing to changes in the temperature, pressure, and gas components. It is important to accurately predict the elemental sulfur solubility in hydrogen sulfide, carbon dioxide, and methane because these are the three main components of high-sulfur-content natural gas. The binary interaction coefficients between sulfur and hydrogen sulfide, carbon dioxide, and methane are the key parameters for predicting the sulfur solubility with a thermodynamic model. In this work, we show that the binary interaction coefficients are not constant, but temperature dependent. Three-parameter temperature-dependent equations for the binary interaction coefficients between sulfur and solvents are proposed. The corresponding regression equations for calculating the binary interaction coefficients between sulfur and hydrogen sulfide, carbon dioxide, and methane are obtained using experimental sulfur solubility data. The average relative errors of the sulfur solubility predicted using the experimental data in hydrogen sulfide, carbon dioxide, and methane using the thermodynamic model with the improved binary interaction coefficients are 6.30%, 1.69%, and 4.34%, and the average absolute relative errors are 7.90%, 13.12%, and 14.98%, respectively. Comparing the improved binary interaction coefficients with four other sets of reported values shows that the solubility values predicted by the thermodynamic model with improved binary interaction coefficients fit the experimental data better.

## Abstract 2

The binary interaction coefficients between sulfur and H_2_S, CO_2_ or CH_4_ are not constant, but temperature dependent. Three-parameter temperature-dependent equations for the binary interaction coefficients between sulfur and solvents are proposed.

## Graphical abstract

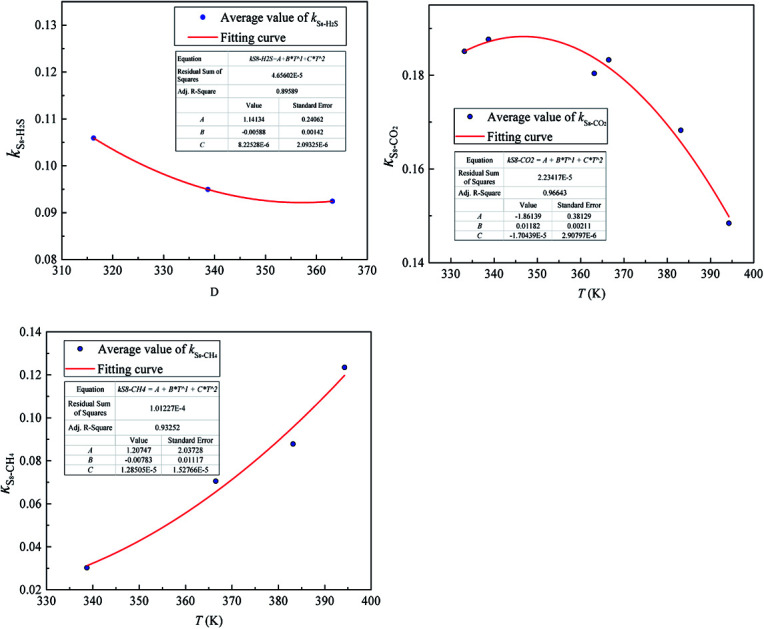

## 1. Introduction

High-sulfur-content natural gas contains hydrogen sulfide, mercaptans, sulfoethers, and other sulfurous substances, with hydrogen sulfide gas comprising the majority of all sulfurous substances. There are many high-sulfur-content gas fields. For example, the hydrogen sulfide volume contents in natural gas mixtures are 15% to 18% in the Puguang Gas Field (China).^1^ High-sulfur-content natural gas fields supply not only clean energy, but also raw materials for sulfur series products.^2^ However, deposited elemental sulfur may cause pore formation, wellbore blockage, and even transmission pipeline blockage and corrosion with changes in pressure, temperature, and gas components.^3–5^ This can lead to the normal production of the gas field being severely inhibited.^6–9^ In recent years, the problem of sulfur deposition has received extensive attention. Notably, elemental sulfur solubility in high-sulfur-content natural gas is the key factor determining whether sulfur deposition occurs.^10–12^

Many researchers have measured the sulfur solubility in hydrogen sulfide (H_2_S), carbon dioxide (CO_2_), and methane (CH_4_), because high-H_2_S-content natural gas mainly comprises these three gas components. Kennedy and Wieland^3^ were the first to measure elemental sulfur solubility in pure H_2_S, CO_2_, CH_4_ and their mixtures in 1960. Their work showed that elemental sulfur solubility increases with increasing pressure and temperature. Roof found that the solubility in H_2_S first increases and then decreases with increasing temperature.^13^ Swift, Brunner, and Gu *et al.* extended the pressure and temperature range for sulfur solubility in H_2_S.^14–16^ Serin *et al.* developed an experimental apparatus for measuring elemental sulfur solubility in CO_2_.^17^ Using the same experimental apparatus, Cloarec *et al.*^18^ obtained experimental data of sulfur solubility in CH_4_.

The above experimental results of solubility in H_2_S, CO_2_, and CH_4_ provide the basis for establishing a solubility predicting model, and strongly support the development of high-sulfur-content natural gas fields.^19–24^ The binary interaction coefficients between sulfur and H_2_S, CO_2_, and CH_4_ in natural gas are important parameters when using a thermodynamic predicting model based on the equation of state (EoS). As shown in Table 1, Heidemann^25^ reported that the binary interaction coefficients between sulfur and H_2_S, CO_2_, or CH_4_ after regression of the experimental data were 0.0812, 0.135, and 0.155, respectively.^25^ In Sun's model,^26^ the binary interaction coefficients between sulfur and H_2_S, CO_2_, and CH_4_ are 0.0758, 0.190, and 0.115, respectively. However, Gu^16^ and Cézac *et al.*^27^ suggested that the binary interaction coefficients are related to the gas mixture temperature. Based on the experimental data of Roof, Brunner, and their own work, Gu^16^ reported binary interaction coefficients between sulfur and H_2_S, CO_2_, and CH_4_ of 316.3–374.8 K, 363.2 and 383.2 K, and 383.2 K, respectively. Cézac *et al.*^27^ proposed three equations for calculating the binary interaction coefficients between sulfur and H_2_S, CO_2_, and CH_4_ based on analysis of experimental data. The binary interaction coefficients from the above studies are considerably different, and it is unclear which set represents better experimental data.

**Table 1:** The binary interaction coefficient values

Comp.	Binary interaction coefficient *k*_*ij*_							Author
S_8_–H_2_S	0.0812							Heidemann R. A.^25^
	0.0758							Sun C. Y.^26^
	0.093–2.079/*T*							Cézac P.^27^
	316.3 K	338.7 K	366.5 K	374.8 K	373.2 K	363.2 K	383.2 K	Gu M. X.^16^
	0.1111	0.1112	0.1042	0.1062	0.1038	0.1033	0.0892	
S_8_–CO_2_	0.135							Heidemann R. A.^25^
	0.190							Sun C. Y.^26^
	0.2423–21.44/*T*							Cézac P.^27^
	363.2 K				383.2 K			Gu M. X.^16^
	0.2107				0.1993			
S_8_–CH_4_	0.155							Heidemann R. A.^25^
	0.115							Sun C. Y.^26^
	1.154–377/*T*							Cézac P.^27^
	0.1345							Gu M. X.^16^

This work aims to evaluate differences in the binary interaction coefficients between sulfur and H_2_S, CO_2_, and CH_4_. We investigated the relationships between the binary interaction coefficients and temperature to extend the range and improve the accuracy of predicting the sulfur solubility in H_2_S, CO_2_, and CH_4_ using a thermodynamic model based on the Peng–Robinson (PR) EoS. Based on experimental data analysis, new three-parameter temperature-dependent equations for calculating the binary interaction coefficients are proposed. The equation parameters are obtained by regression analysis of the experimental data. Furthermore, we compared the solubility results calculated using the thermodynamic model with the binary interaction coefficients reported by Heidemann, Sun, Cézac, Gu, and in this work.

## 2. Model description

### 2.1. Governing equations

The model is based on thermodynamic gas–solid phase equilibrium theory, which assumes perfect mixing of the sulfur and gas components. As mentioned by Gu, Sun, and Heidemann, solid sulfur should be treated as single molecule S_8_.^16,25,26^ The phase equilibrium condition of the gas–solid system requires the fugacity of gaseous sulfur to be the same as that of the solid phase, as expressed by eqn (1):^28^1
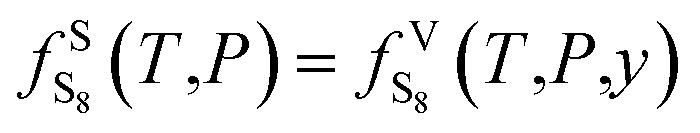


### 2.2. Solid phase fugacity

The solid sulfur fugacity is related to the saturation vapor pressure of solid sulfur, as expressed by eqn (2):^28^2
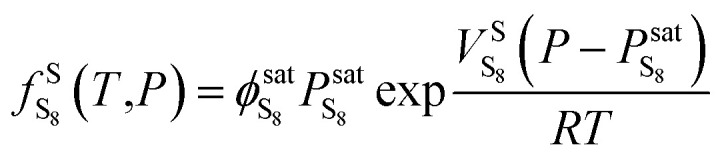


The sulfur saturation vapor pressure is always small in the gas–solid phase equilibrium system, so 
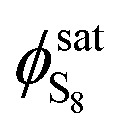
 = 1.0 in this model.^28^ Shuai and Meisen^29^ reported regression equations of the sulfur saturation vapor pressure at different temperatures.

When *T* < 368 K, 
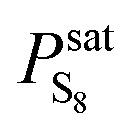
 is expressed by eqn (3): 3
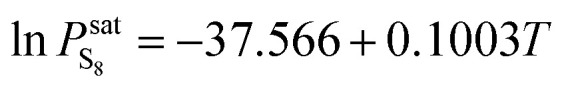


When *T* > 368 K, 
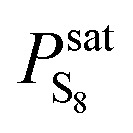
 is expressed by eqn (4):4
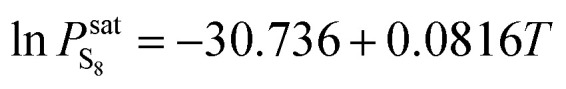
5
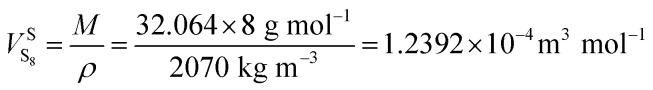


Using eqn (2)–(5), we can calculate the fugacity of sulfur in solid phase.

### 2.3. Elemental sulfur fugacity in the gas phase

The elemental sulfur fugacity in the gas phase can be expressed with eqn (6):^28^6
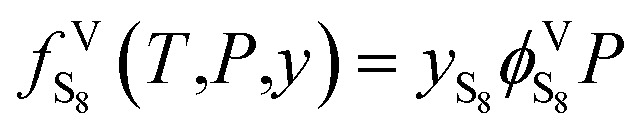


#### 2.3.1. Peng–Robinson (PR) EoS

According to eqn (6), 
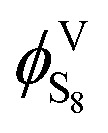
 is the key parameter for calculating the gaseous sulfur fugacity, and can be described by the PR EoS. The basic form of the PR EoS is shown in eqn (7):^30^7
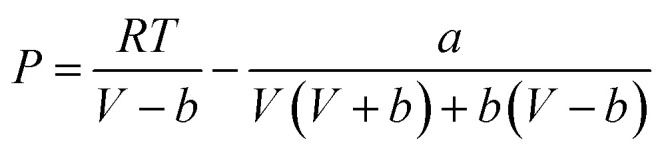
where *a* and *b* are the parameters of PR EoS. When gas mixtures are single component, *a* and *b* can be expressed as follows:8
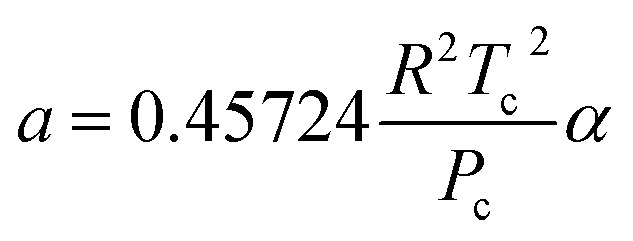
9
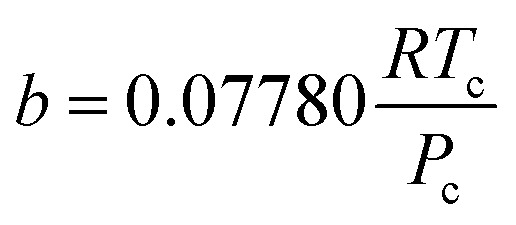

10 *α* = [1 + (1 − *T*_r_^0.5^)(0.37464 + 1.54226*ω* − 0.26992*ω*^2^)]^2^

11
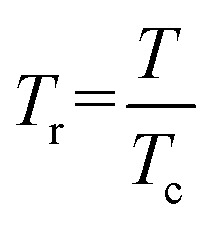


Table 2 shows the critical parameters and acentric factors of S_8_, H_2_S, CO_2_, and CH_4_.^27,28^

**Table 2:** Critical parameters and acentric factors

Component	Critical pressure (MPa)	Critical temperature (K)	Acentric factor
S_8_	5.2	1065.0	0.3805
H_2_S	8.963	373.5	0.094
CO_2_	7.383	304.2	0.224
CH_4_	4.599	190.6	0.012

After the solid and gas phase sulfur reach the equilibrium state, the gas phase is composed of a solvent gas component and gaseous sulfur. The *a* and *b* parameters need be calculated using the mixing rule according to the values of the single components. In this model, *b* is calculated by the classic mixing rule, as follows:12
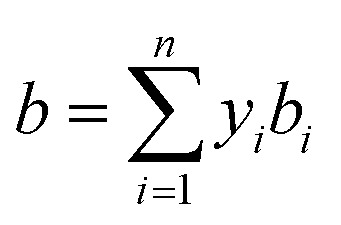


The *a* parameter of the gas mixture components will be discussed in detail in Section 3.

The fugacity coefficient of gaseous sulfur 
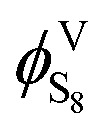
 can be expressed as:13
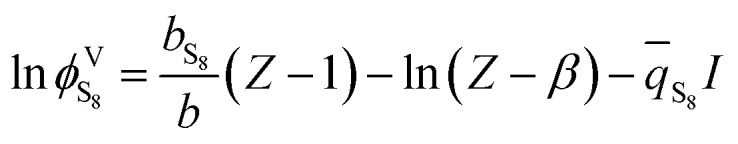
where14
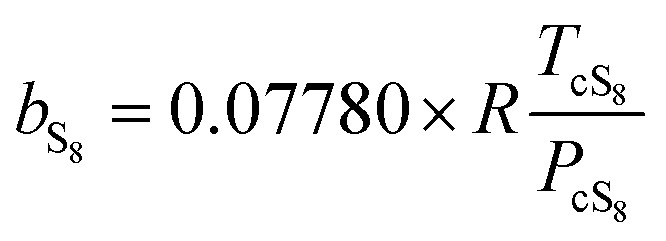
*Z* is calculated using15
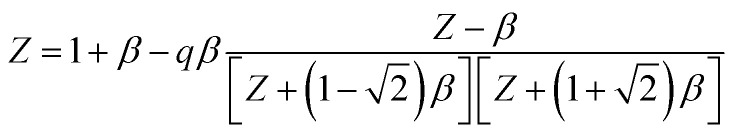


Relevant parameters in eqn (15) are expressed by eqn (16)–(19):16
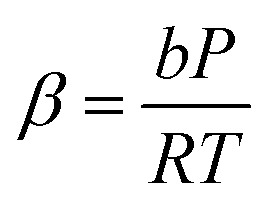
17
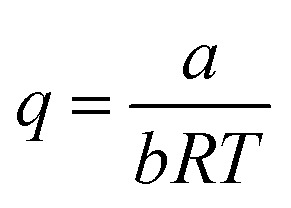
18
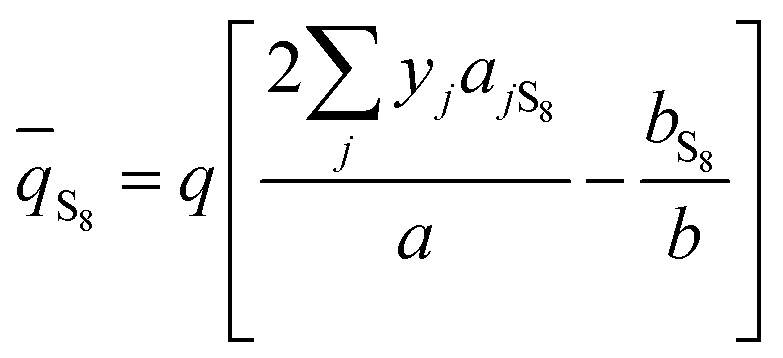
19
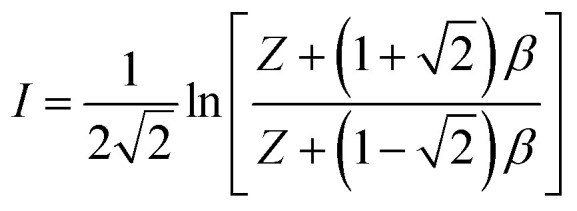


In this work, this equation was solved using the Newton–Raphson method. The elemental sulfur solubilities in H_2_S, CO_2_, and CH_4_ can be calculated easily using eqn (1)–(19).

## 3. Proposed method

The classical mixing rule for *a* is:^28^20
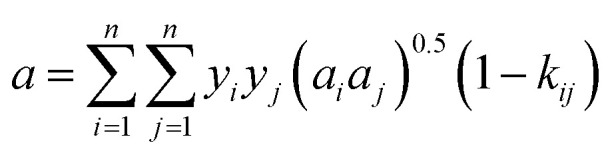
where *k*_*ij*_ are the binary interaction coefficients between S_8_ and the gas solvents (H_2_S, CO_2_, and CH_4_). Eqn (20) shows that *k*_*ij*_ are important parameters.

The reported binary interaction coefficients between S_8_ and H_2_S, CO_2_, and CH_4_ obtained by regression of the sulfur solubility experimental data are shown in Table 1. The binary interaction coefficients reported by Sun and Heidemann are constant.^25,26^ However, as mentioned by Gu^16^ and Cézac,^27^ the binary interaction coefficients are temperature dependent. Therefore, we proposed a new mixing rule for *a* to calculate the gaseous S_8_ fugacity:21
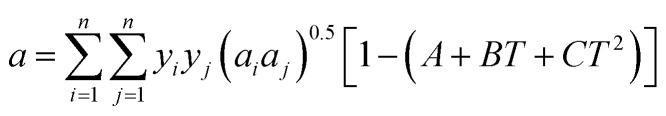


Therefore,

22 *k*_*ij*_ = *A* + *BT* + *CT*^2^

**Fig. 1:**
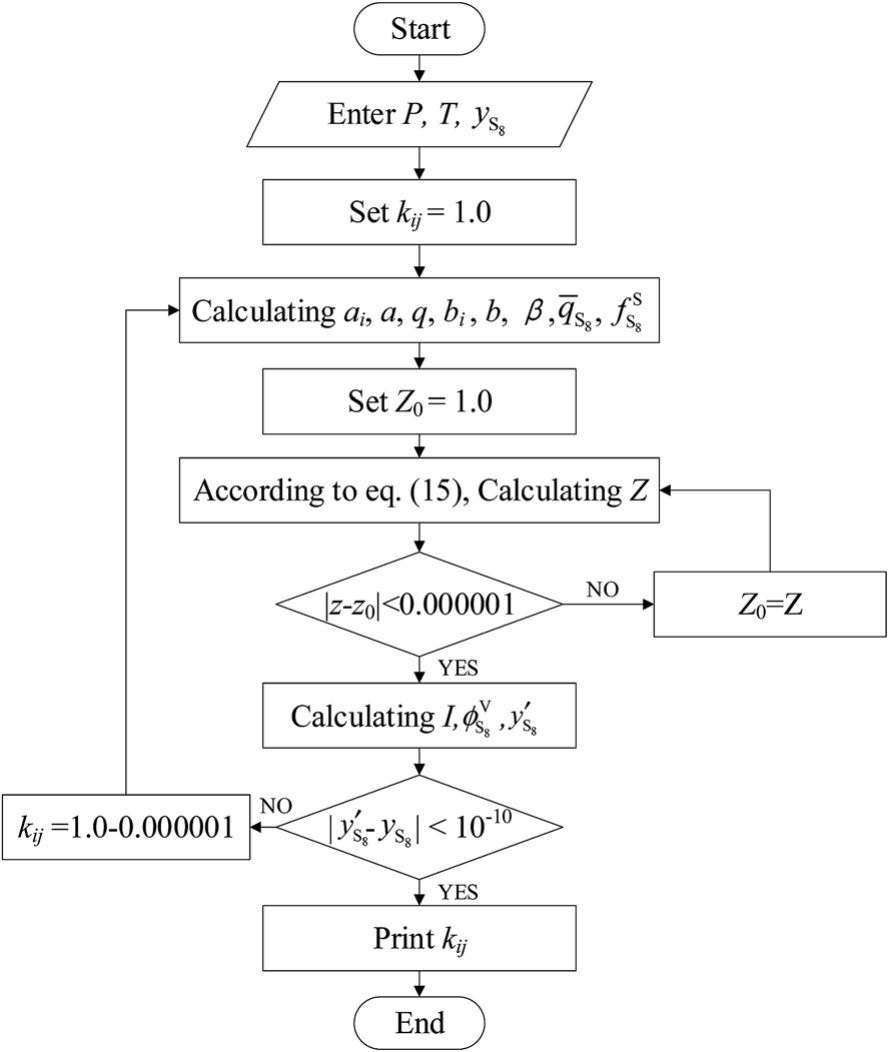
Calculating diagram of *k*_S_8_*j*_.

The *k*_*ij*_ values with temperature were regressed based on the experimental data of Kennedy, Roof, Brunner, Serin, and Gu,^3,13,15–17^ and the calculated diagram is shown in Fig. 1.

**Fig. 2:**
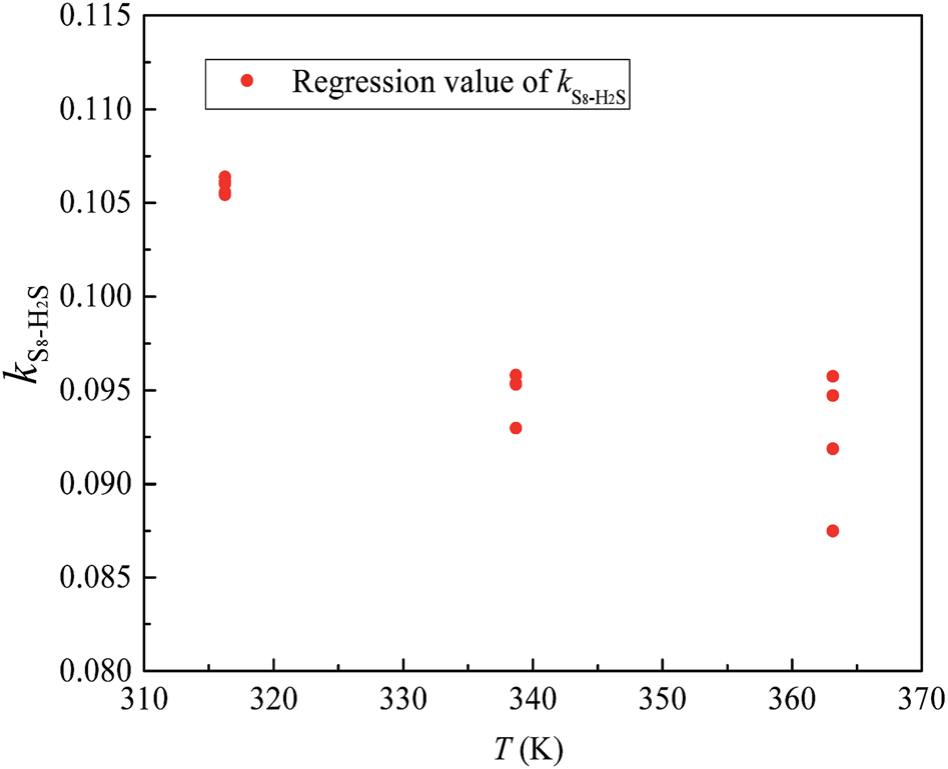
The *k*_*ij*_ between S_8_ and H_2_S.

**Fig. 3:**
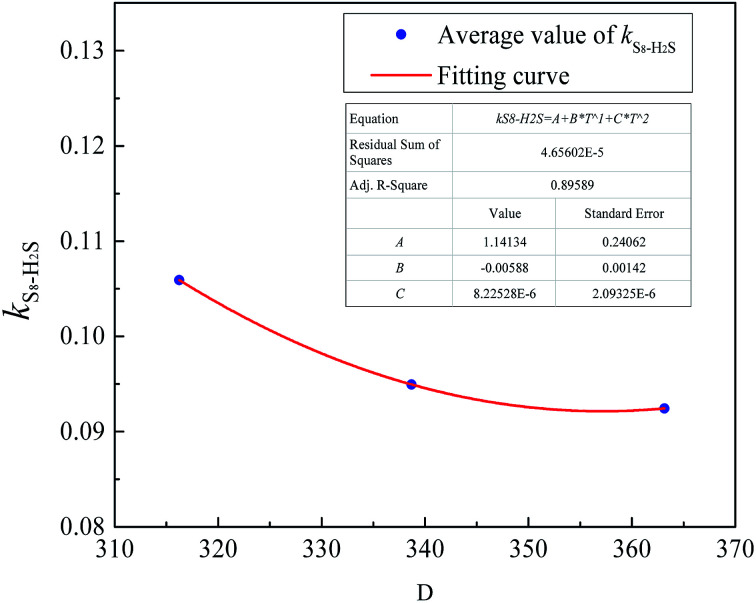
The *k*_*ij*_ fitting curve between S_8_ and H_2_S.

According to Eslamimanesh's study of the consistency of the experimental results of sulfur solubility in H_2_S, only ∼45% of the values are reliable.^20^ To obtain an equation for the binary interaction coefficient between sulfur and H_2_S, we chose the 14 sets of experimental data at temperatures of 316.26, 338.71, and 363.15 K reported by Roof and Gu.^13,16^Fig. 2 shows the binary interaction coefficient between S_8_ and H_2_S calculated at temperatures of 316.26–363.15 K. To better fit the *k*_*ij*_ value, we obtained the average *k*_*ij*_ values at the same temperatures shown in Fig. 3, and a new equation and fitting curve of *k*_S_8_–H_2_S_ with temperature were obtained. The adjusted *R*^2^ value (*R*_adj_^2^) was 0.896, which showed that the fitting precision was high. The new equation for the binary interaction coefficient between S_8_ and H_2_S is:23
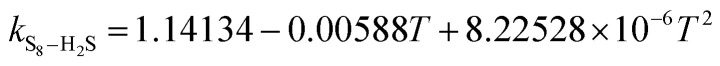


**Fig. 4:**
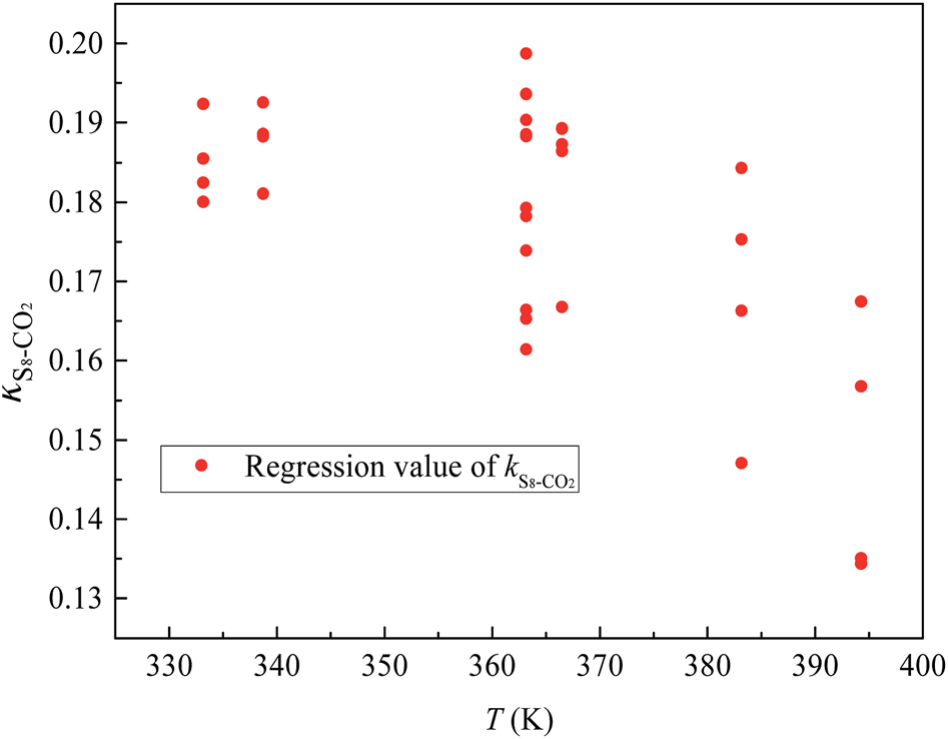
The *k*_*ij*_ between S_8_ and CO_2_.

**Fig. 5:**
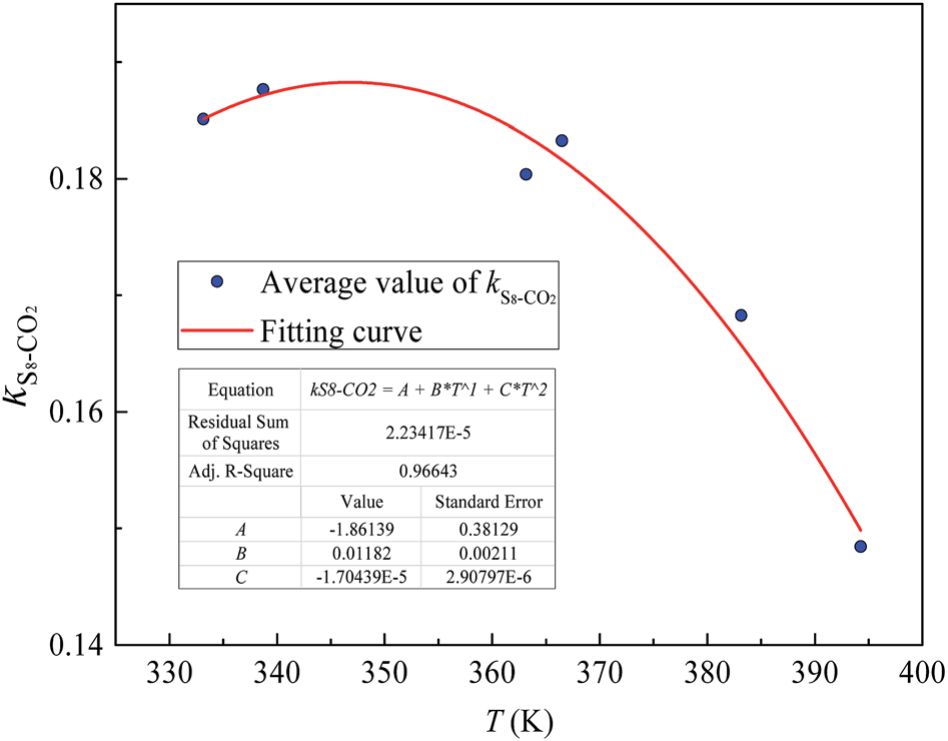
The *k*_*ij*_ fitting curve between S_8_ and CO_2_.

Fig. 4 and 5 show the new equation and fitting curve for *k*_S_8_–CO_2__ in the temperature range 333.15–394.26 K (*R*_adj_^2^ = 0.966). The new equation for the binary interaction coefficient between S_8_ and CO_2_ is:24
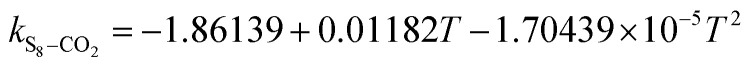


**Fig. 6:**
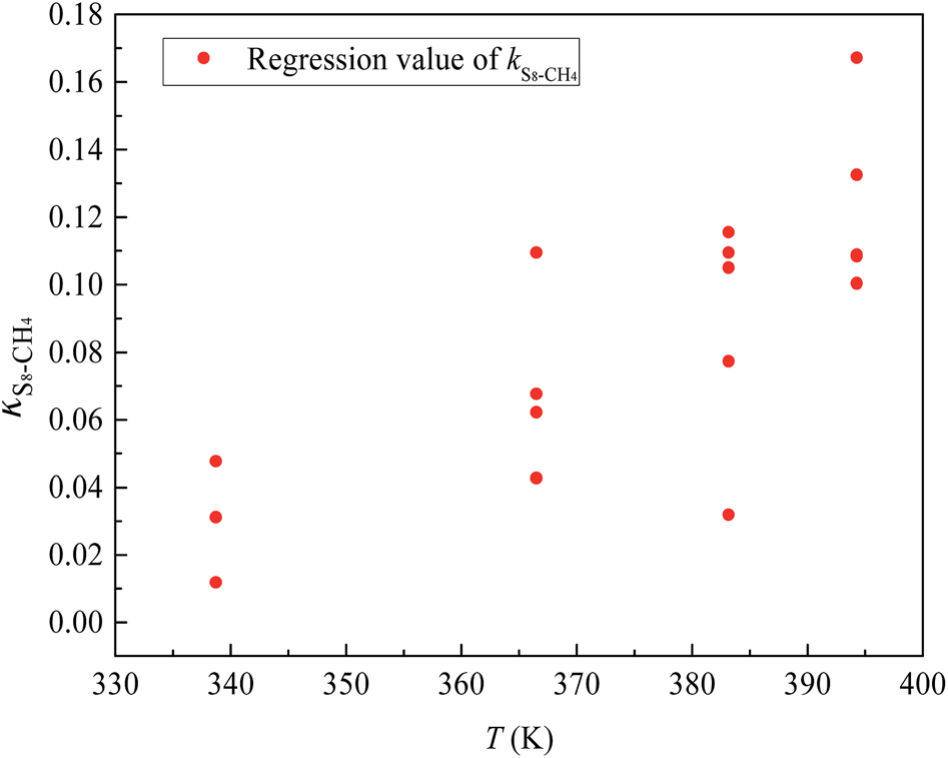
The *k*_*ij*_ between S_8_ and CH_4_.

**Fig. 7:**
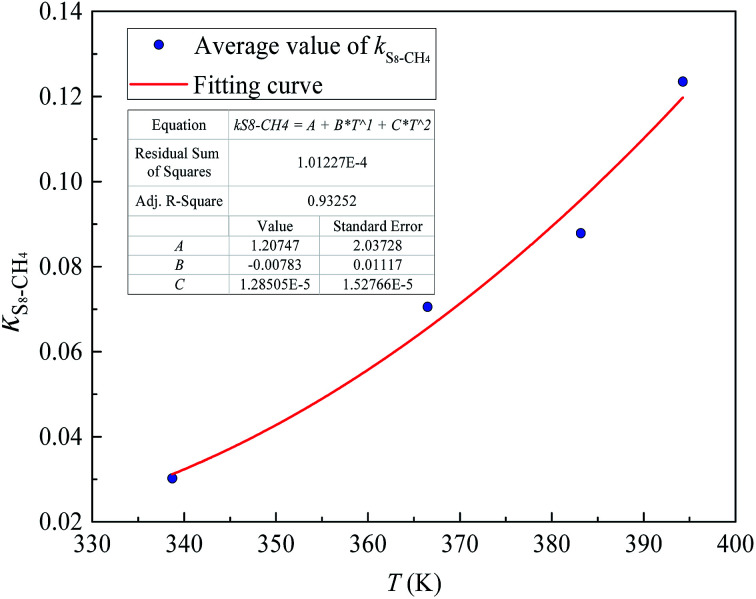
The *k*_*ij*_ fitting curve between S_8_ and CH_4_.

Fig. 6 and 7 show the new equation and fitting curve for *k*_S_8_–CH_4__ in the temperature range 338.71–394.26 K (*R*_adj_^2^ = 0.933). The new equation for the binary interaction coefficient between S_8_ and CH_4_ is:25
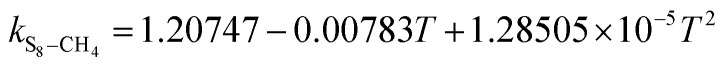


## 4. Results and discussion

### 4.1. Predicting the sulfur solubility using the proposed binary interaction coefficients

The sulfur solubility in H_2_S predicted by the thermodynamic model using the proposed binary interaction coefficient between sulfur and H_2_S obtained by eqn (23) is shown in Table 5, along with the experimental results of Roof and Gu at temperatures of 316.26, 338.71, and 363.15 K and in the pressure range 7.03–32.03 MPa.^13,16^ Parameter *a* in this model was calculated with the proposed mixing rule (eqn (21)) and (23).

**Table 3:** Deviation comparisons of predicting solubility in CO_2_ with different *k*_S_8_–CO_2__

Deviation	*k*_S_8_–CO_2__					
	Improved *k*_S_8_–CO_2__	0.190	0.135	363.2 K	383.2 K	0.2423–21.44/*T*
				0.2107	0.1993	
ARE (%)	1.69	−14.57	111.32	−34.20		−3.11
AARE (%)	13.12	16.38	111.36	34.20		18.22

**Table 4:** Deviation comparisons of predicting solubility in CH_4_ with different *k*_S_8_–CH_4__

Deviation	*k*_S_8_–CH_4__				
	Improved *k*_S_8_–CH_4__	0.115	0.155	383.2 K	1.154–377/*T*
				0.1345	
ARE (%)	4.34	−20.08	−40.70	−26.58	−33.04
AARE (%)	14.98	25.23	41.66	26.58	34.07

There are 14 sets of experimental data in Table 5. The RE, ARE, and AARE values were calculated using eqn (26)–(28):26
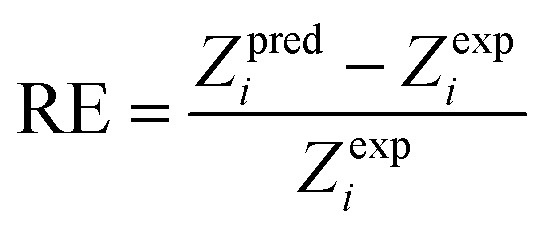
27
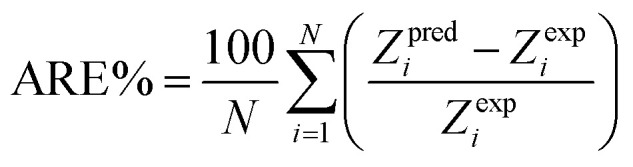
28
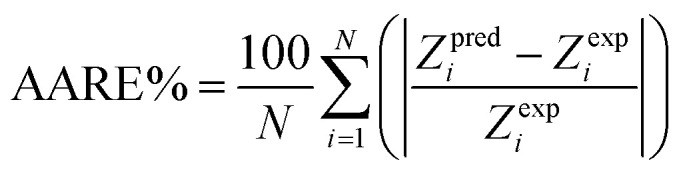


Based on Table 5, the total ARE and AARE values for predicting the sulfur solubility were 6.30% and 7.90%, respectively.

**Fig. 8:**
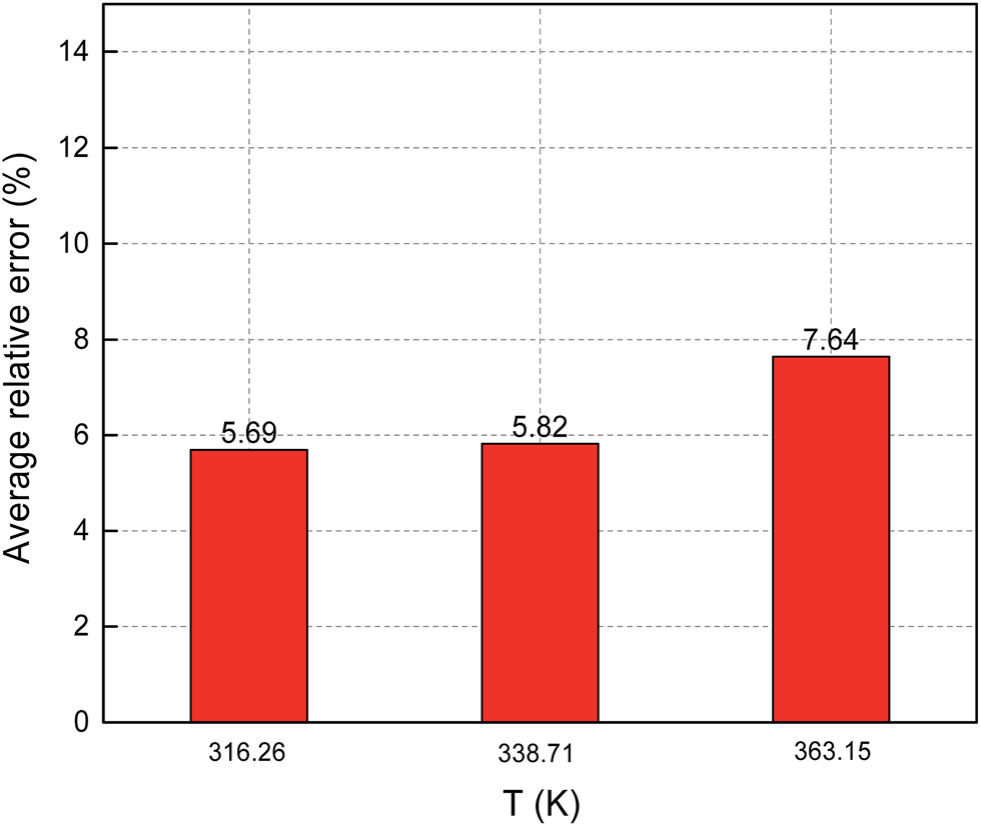
ARE of sulfur solubility in H_2_S.

**Fig. 9:**
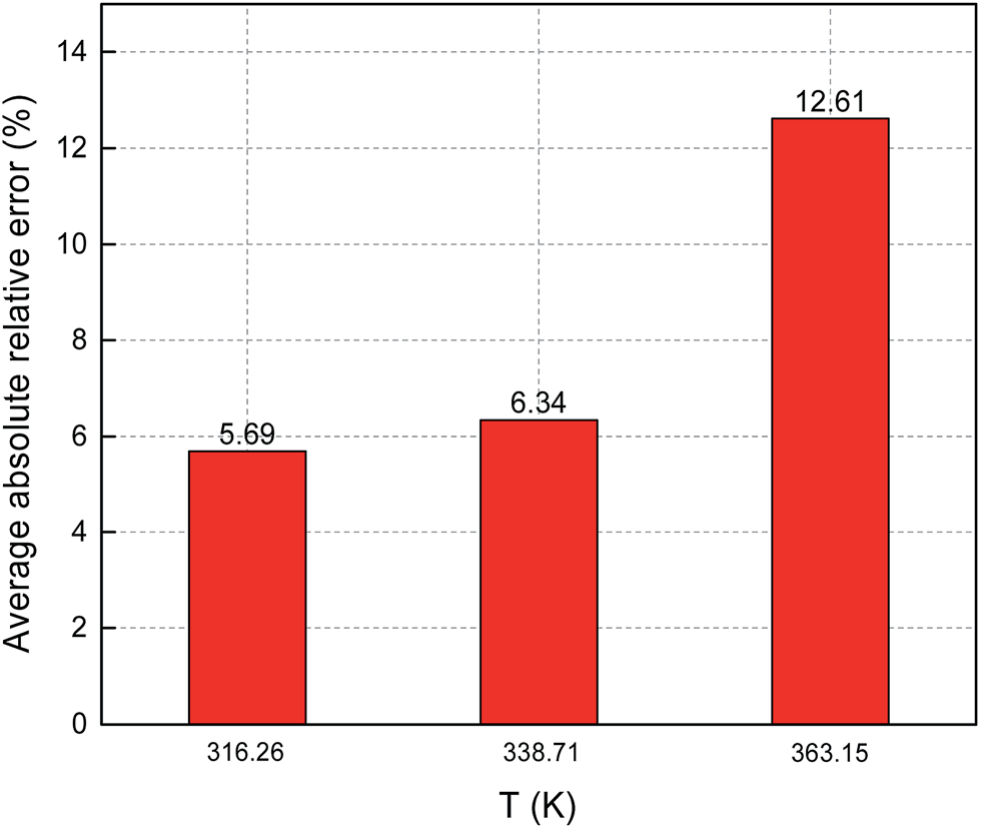
AARE of sulfur solubility in H_2_S.

Fig. 8 and 9 show the ARE and AARE values of sulfur solubility in H_2_S calculated from the experimental data at three different temperatures (316.26, 338.71, and 363.15 K). Fig. 8 shows that the ARE values were positive at temperatures of 316.26, 338.71, and 363.15 K. The lowest ARE value was 5.69% at 316.26 K, with a highest ARE value of 7.64% at 363.15 K. Fig. 9 shows that the greatest AARE value was 12.61% at 363.15 K. The other AARE values were all below 7%. Considering the critical temperature of H_2_S (373.5 K), the large deviation at 363.15 K could be due to the kinetic characteristics of the solvent molecule (H_2_S) being influenced by external factors near the critical temperature and pressure.^16^

**Fig. 10:**
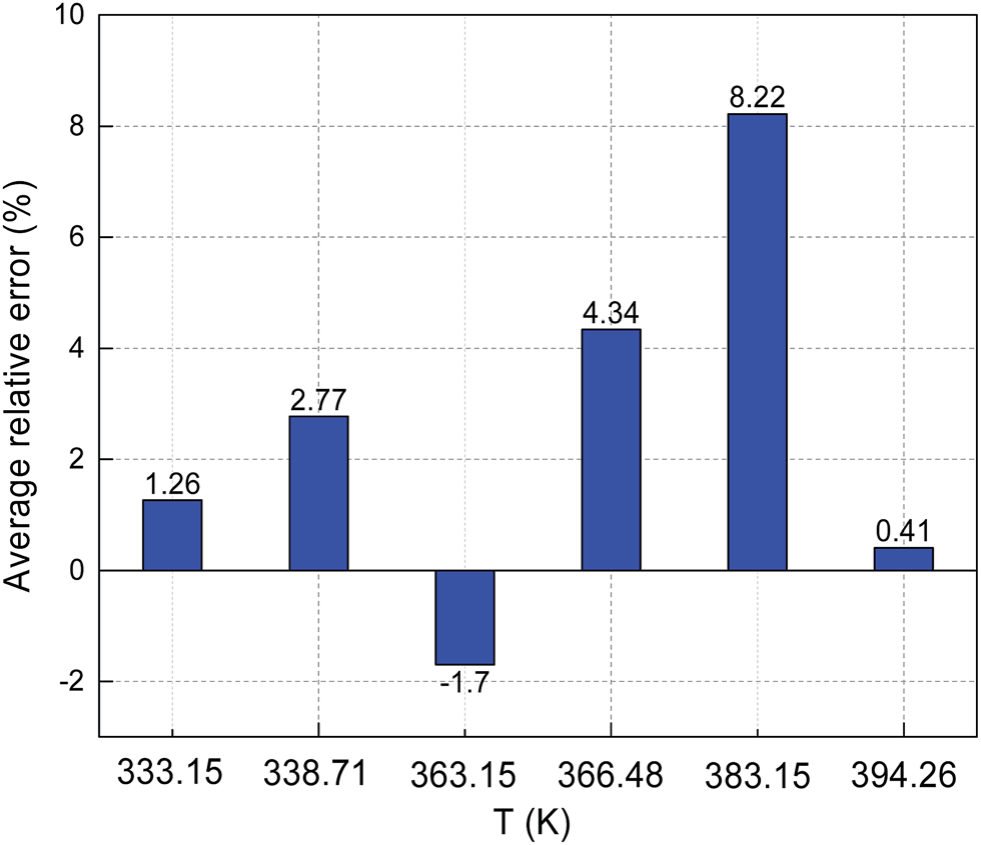
ARE of sulfur solubility in CO_2_.

**Fig. 11:**
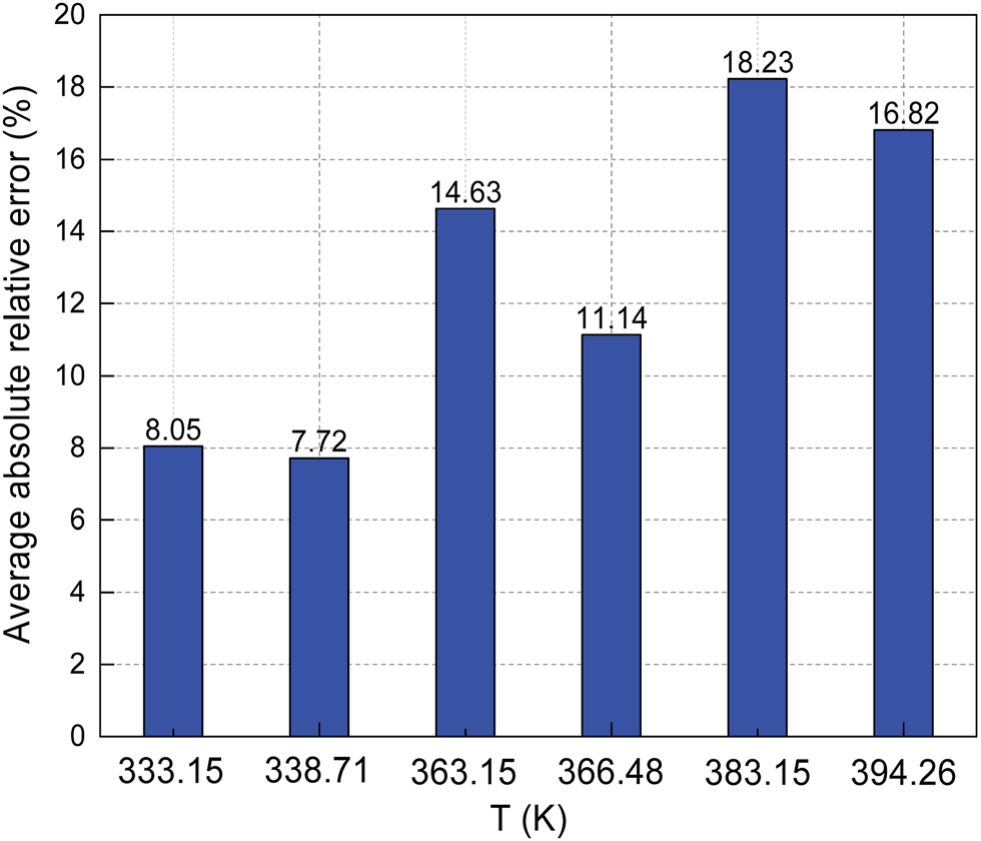
AARE of sulfur solubility in CO_2_.

The predicted sulfur solubility in CO_2_ using the proposed binary interaction coefficient, along with the experimental data of Kennedy, Gu, and Serin, in the temperature range 333.15–394.26 K at pressures of 15.1–41.4 MPa are shown in Table 6.^3,16,17^ Parameter *a* with the proposed mixing rule (eqn (21)) was calculated using eqn (24). There were 32 sets of experimental data included in Table 6. Based on these calculated results, the total ARE and AARE values of the predicted sulfur solubility in CO_2_ were 1.69% and 13.12%, respectively. Fig. 10 and 11 show the ARE and AARE values of sulfur solubility in CO_2_ calculated using the experimental data for six temperature ranges from 333.15–394.26 K. The lowest ARE value was 0.41% at 394.26 K, and the highest ARE value was 8.22% at 383.15 K (Fig. 10). Fig. 11 shows that the highest AARE value was 18.23% at 383.15 K.

**Fig. 12:**
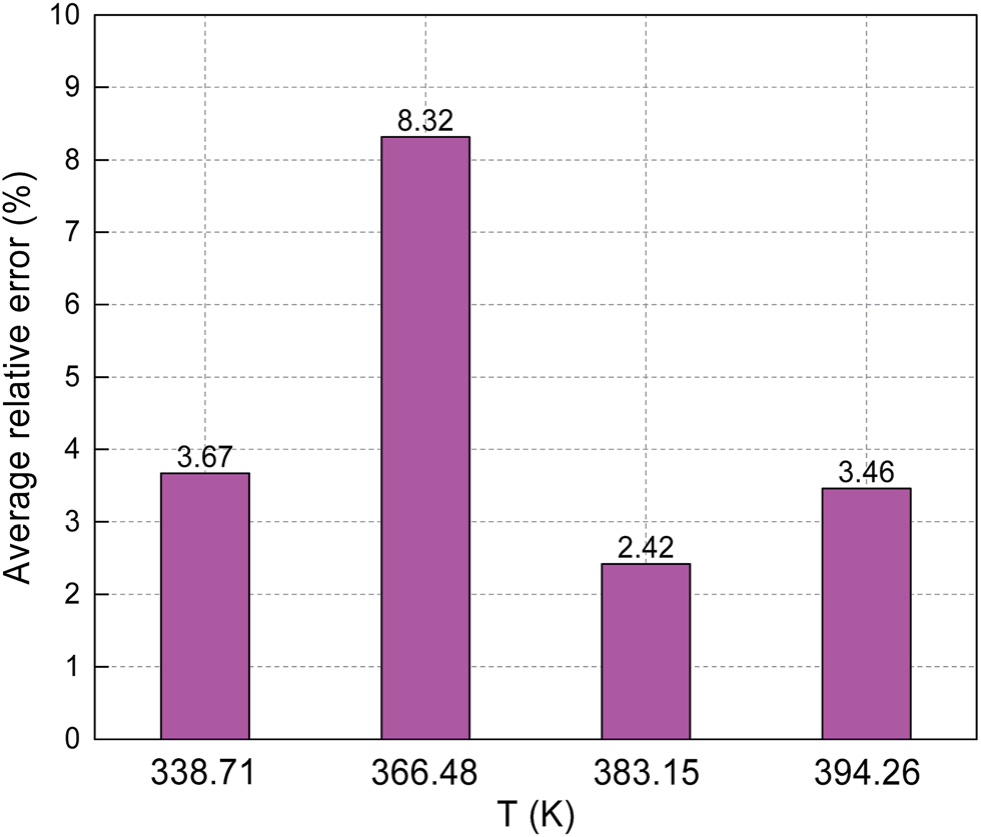
ARE of sulfur solubility in CH_4_.

**Fig. 13:**
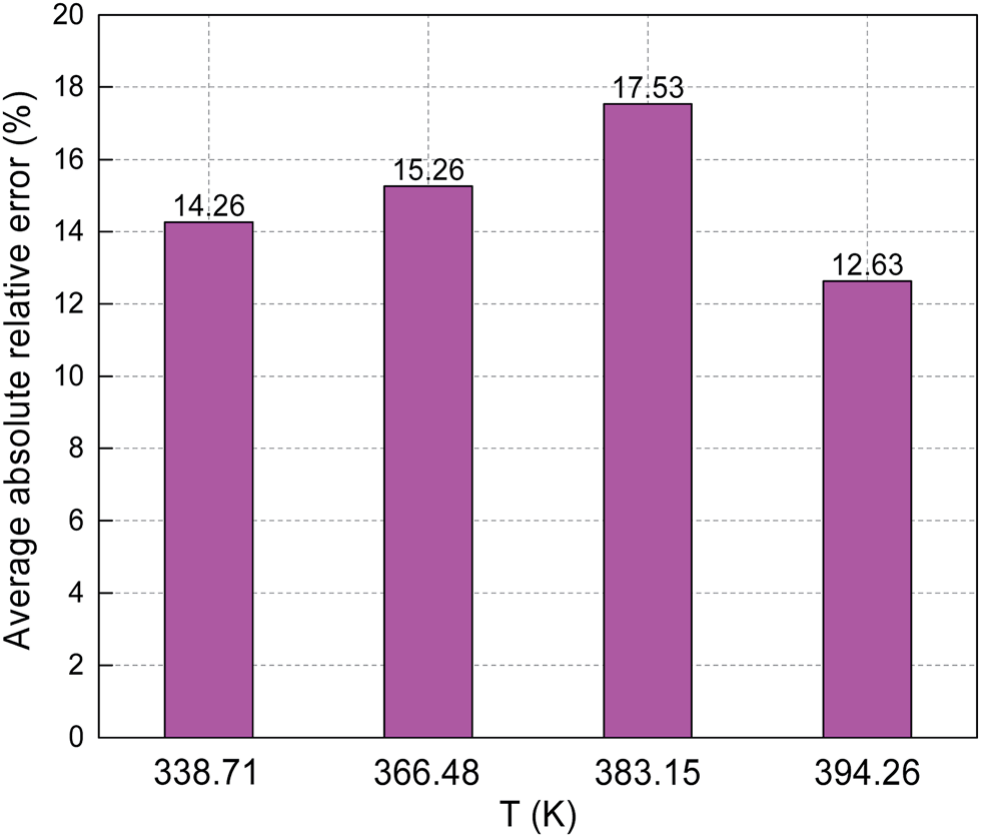
AARE of sulfur solubility in CH_4_.

The predicted sulfur solubility in CH_4_ using the proposed binary interaction coefficient, along with the experimental data of Kennedy and Gu in the temperature range 338.71–394.26 K and pressures range 6.89–41.4 MPa, are shown in Table 7.^3,16^ Parameter *a* with the proposed mixing rule (eqn (21)) was calculated with eqn (25). There are 17 sets of experimental data in Table 7. Based on the calculated results in Table 7, the total ARE and AARE values calculated with eqn (27) and (28) were 4.34% and 14.98%, respectively. Fig. 12 and 13 show the ARE and AARE values of sulfur solubility in CH_4_ calculated using the experimental data for four temperatures in the range 338.71–394.26 K. Fig. 12 shows that the lowest ARE value was 2.42% at 383.15, while the highest ARE value was 8.32% at 366.48 K. The highest AARE value was 17.53% at 383.15 K (Fig. 13). Combined with the results of the calculated sulfur solubilities in CO_2_ and CH_4_, the main reason for the large deviation could be that the solubility values in CO_2_ and CH_4_ are too small.

### 4.2. Comparison of binary interaction coefficients

#### 4.2.1. Comparison of sulfur solubility in H_2_S with different *k*_S_8_–H_2_S_

**Fig. 14:**
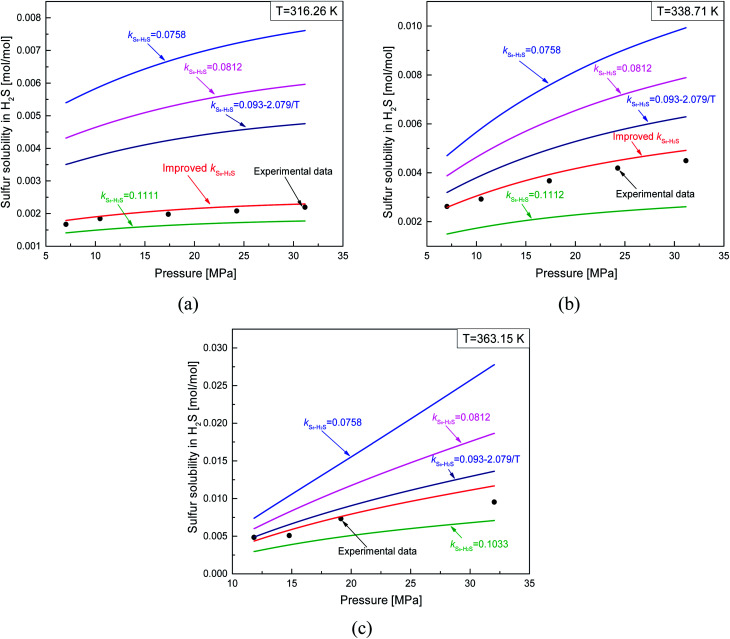
Comparisons of predicting sulfur solubility in H_2_S with different *k*_S_8_–H_2_S_.

As mentioned above, Sun and Heidemann suggested that *k*_S_8_–H_2_S_ is temperature independent.^15,16^ However, Gu and Cézac *et al.* suggested that *k*_S_8_–H_2_S_ is temperature dependent.^16,27^ Gu determined *k*_S_8_–H_2_S_ values at different temperatures from 316.3 to 383.2 K based on experimental data, and Cézac proposed a temperature-dependent *k*_S_8_–H_2_S_ equation.^16,27^ Here, we compared the sulfur solubilities predicted using these different *k*_S_8_–H_2_S_ values with our proposed *k*_S_8_–H_2_S_ model (eqn (23)). Fig. 14 shows that the accuracy of the predicted sulfur solubility in H_2_S with the proposed *k*_S_8_–H_2_S_ was significantly higher than those of the other four *k*_S_8_–H_2_S_ values. At 316.26 K, the value proposed by Gu was more reasonable than the three other values. The deviation in the solubility calculated with the thermodynamic model and the experimental data was relatively large using the values reported by Sun, Heidemann, and Cézac. The three values also showed that the predicted solubility was closely related to the binary interaction coefficient between sulfur and H_2_S. Furthermore, for the sulfur solubility in H_2_S, the model with the proposed *k*_S_8_–H_2_S_ applied to temperatures ranging from 316.26 to 363.15 K and pressures ranging from 7.03 to 32.03 MPa.

#### 4.2.2. Comparison of sulfur solubility in CO_2_ with different *k*_S_8_–CO_2__

**Fig. 15:**
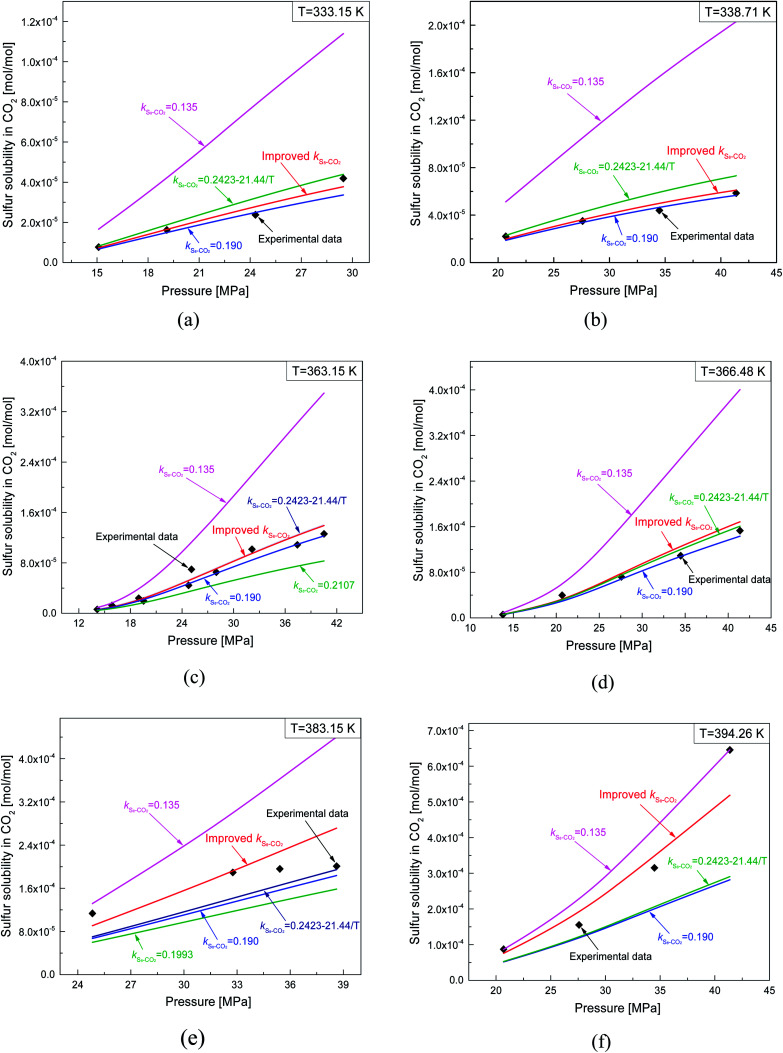
Comparisons of predicting sulfur solubility in CO_2_ with different *k*_S_8_–CO_2__.

Fig. 15 shows a comparison of the sulfur solubility in CO_2_ calculated using the thermodynamic model with five different *k*_S_8_–CO_2__ values at temperatures ranging from 333.15 to 394.26 K. When *k*_S_8_–CO_2__ was 0.135, the difference between the calculated sulfur solubility and the experimental data was the largest at 383.15 K. Based on Fig. 15, the accuracy of the predicted sulfur solubility seemed to be acceptable using all of the other four *k*_S_8_–CO_2__ values except 0.135.

As shown in Table 1, the *k*_S_8_–CO_2__ values obtained by Sun and Heidemann were 0.190 and 0.135.^25,26^ Gu *et al.*^16^ related the *k*_S_8_–CO_2__ parameter to the temperature, and reported *k*_S_8_–CO_2__ values of 0.2107 and 0.1993 at 363.2 and 383.2 K.^16^ Cézac^27^ suggested that *k*_S_8_–CO_2__ was temperature dependent and proposed an equation for calculating *k*_S_8_–CO_2__, as shown in Table 1. A comparison of the total ARE and AARE values of the calculated sulfur solubility in CO_2_ with different *k*_S_8_–CO_2__ values is shown in Table 3. From Table 3, the deviations of the solubility values calculated using the proposed *k*_S_8_–CO_2__ from the experimental data were smaller than those using the other four *k*_S_8_–CO_2__ values.

**Fig. 16:**
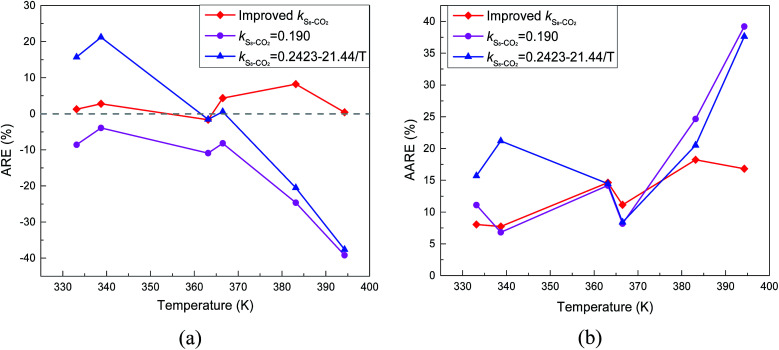
The deviation of predicting solubility in CO_2_ with different *k*_S_8_–CO_2__.

A comparison of the ARE and AARE values for different *k*_S_8_–CO_2__ at temperatures of 333.15 to 394.26 K is shown in Fig. 16. Fig. 16(a) shows that ARE is the closest to 0% with the proposed *k*_S_8_–CO_2__, ARE < 0 when *k*_S_8_–CO_2__ is 0.190, and ARE is both negative and positive for the *k*_S_8_–CO_2__ reported by Cézac.^27^ Using the *k*_S_8_–CO_2__ and *k*_S_8_–CO_2__ values reported by Cézac,^27^ the ARE values were −1.67% and −1.57% at 363.15 K, and 4.34% and 0.55% at 366.48 K, respectively.

As shown Fig. 16(b), the AARE values were smaller using the proposed *k*_S_8_–CO_2__ than using the other two *k*_S_8_–CO_2__ values. At 338.71 K, the AARE values were 7.72% and 6.82% using the proposed *k*_S_8_–CO_2__ and *k*_S_8_–CO_2__ = 0.190, respectively. Using the proposed *k*_S_8_–CO_2__, the *k*_S_8_–CO_2__ reported by Sun, and the *k*_S_8_–CO_2__ reported by Cézac, the AARE values were 14.63%, 14.18%, and 14.64% at 363.15 K, and 11.14%, 8.18%, and 8.40% at 366.48 K, respectively. Fig. 16 shows that the accuracy of the predicted solubility using *k*_S_8_–CO_2__ reported by Cézac was higher than that using the *k*_S_8_–CO_2__ proposed in this paper at 363.15 and 366.48 K. However, the total deviation was still the lowest using the proposed binary interaction coefficient between sulfur and CO_2_ (Table 6 and Fig. 16).

#### 4.2.3. Comparison of sulfur solubility in CH_4_ with different *k*_S_8_–CH_4__

As shown in Table 1, the *k*_S_8_–CH_4__ values reported by Heidemann and Sun are 0.155 and 0.115, respectively.^25,26^ Gu *et al.* related *k*_S_8_–CH_4__ to temperature, but only reported the *k*_S_8_–CH_4__ value at 383.2 K.^16^ Cézac *et al.*^27^ proposed a temperature-dependent equation for calculating *k*_S_8_–CH_4__ (Table 1). The total ARE and AARE values of the calculated sulfur solubility in CH_4_ using the different values of *k*_S_8_–CH_4__ are shown in Table 4. The lowest ARE and AARE values of 4.34% and 14.98% were obtained using the proposed *k*_S_8_–CH_4__. When *k*_S_8_–CH_4__ = 0.155, the deviations between the calculated solubility values and the experimental data were the largest. From Table 4, the deviations between the solubility values calculated using the proposed *k*_S_8_–CH_4__ and the experimental data were smaller than those using the other four *k*_S_8_–CH_4__.

**Fig. 17:**
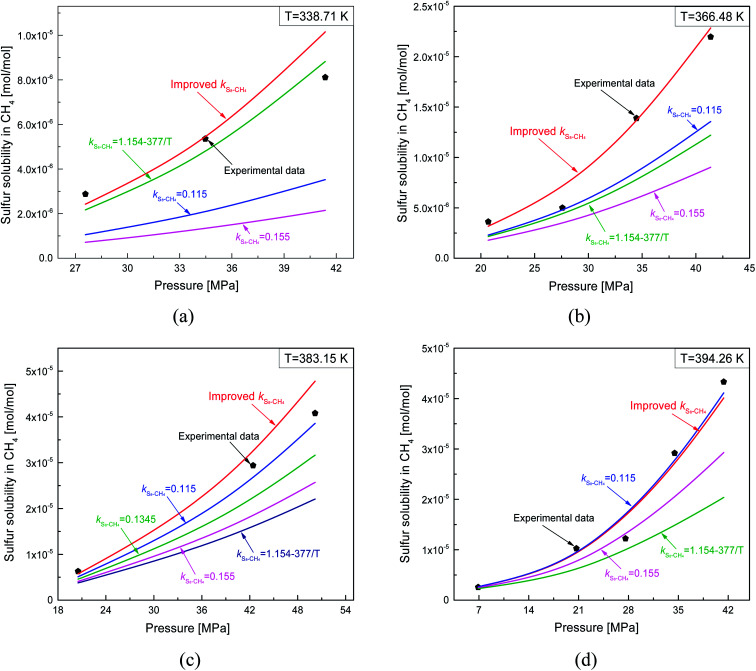
Comparisons of predicting sulfur solubility in CH_4_ with different *k*_S_8_–CH_4__.

Fig. 17 shows a comparison of the predicted sulfur solubility in CH_4_ using the thermodynamic model with five different *k*_S_8_–CH_4__ at temperatures ranging from 338.71 to 394.26 K. Fig. 17(c) shows the calculated sulfur solubility in CH_4_ with five different *k*_S_8_–CH_4__ at 383.15 K, because *k*_S_8_–CH_4__ = 0.1345 reported by Gu was only suitable at 383.2 K. Therefore, Fig. 17(a), (b), and (d) only show the predicted solubility with four different *k*_S_8_–CH_4__.

As shown in Fig. 17, the predicted solubility values were negative, except for that calculated using the proposed *k*_S_8_–CH_4__. When *k*_S_8_–CH_4__ = 0.115 at 394.26 K, the calculated solubility in CH_4_ was close to the experimental data (Fig. 17(d)). Using *k*_S_8_–CH_4__ = 0.115 and the proposed *k*_S_8_–CH_4__, the ARE values were 5.25% and 3.46%, while the AARE values were 12.11% and 12.63%, respectively. This indicated that the predicted solubility at 394.26 K using the *k*_S_8_–CH_4__ value reported by Sun was also acceptable.^26^

## 5. Conclusions

We have proposed new three-parameter equations to calculate the binary interaction coefficients between sulfur and H_2_S, CO_2_, and CH_4_. The parameters in these three equations were obtained by regression of the experimental sulfur solubility data in H_2_S, CO_2_, and CH_4_. The relationship between the binary interaction coefficients and temperature was quadratic. The binary interaction coefficients *k*_S_8_–H_2_S_, *k*_S_8_–CO_2__, and *k*_S_8_–CH_4__ were expressed by eqn (23)–(25), and the *R*_adj_^2^ values of the new fitting equations were 0.896, 0.966, and 0.933, respectively, which indicated that the new three-parameter equations were reliable.

By comparing the experimental solubility data with the solubility results predicted using the binary interaction coefficients proposed by this work and those reported by Sun, Heidemann, Gu, and Cézac, the total ARE and AARE values were significantly smaller when using the proposed binary interaction coefficients than those when using the other four. However, the accuracy of the predicted sulfur solubility in CO_2_ using *k*_S_8_–CO_2__ reported by Cézac was slightly higher than that using *k*_S_8_–CO_2__ proposed in this article at temperatures of 363.15 and 366.48 K. Furthermore, the calculated solubility in CH_4_ using *k*_S_8_–CH_4__ reported by Sun was acceptable.

In general, the predicted results were satisfactory considering the ARE and AARE values obtained using the proposed binary interaction coefficients. The accuracies of the predicted sulfur solubilities in H_2_S, CO_2_, and CH_4_ using the thermodynamic model with the proposed binary interaction coefficients based on the PR EoS were greatly improved, and the thermodynamic model was suitable for a wide range of temperatures and pressures.

## Conflicts of interest

There are no conflicts to declare.

## Appendix

**Table 5:** Elemental sulfur solubility in H_2_S

*T*/K	*P*/MPa	Solubility (experiment)/mol mol^−1^	Solubility (this model)/mol mol^−1^	Relative error
316.26	7.03	0.001669	0.001790	0.0722
	10.48	0.001846	0.001915	0.0373
	17.37	0.001977	0.002099	0.0619
	24.27	0.002078	0.002220	0.0682
	31.16	0.002196	0.002294	0.0448
338.71	7.03	0.00262	0.002586	−0.0129
	10.48	0.002926	0.003120	0.0662
	17.37	0.00367	0.003927	0.0701
	24.27	0.004189	0.004502	0.0747
	31.16	0.004494	0.004913	0.0932
363.15	11.83	0.004832	0.004352	−0.0993
	14.79	0.005081	0.005789	0.1393
	19.14	0.007313	0.007613	0.0410
	32.03	0.009523	0.011664	0.2248

**Table 6:** Elemental sulfur solubility in CO_2_

*T*/K	*P*/MPa	Solubility (experiment)/mol mol^−1^	Solubility (this model)/mol mol^−1^	Relative error
333.15	15.10	7.682 × 10^−6^	7.400 × 10^−6^	−0.0368
	19.10	1.624 × 10^−5^	1.650 × 10^−5^	0.0144
	24.31	2.377 × 10^−5^	2.790 × 10^−5^	0.1718
	29.47	4.193 × 10^−5^	3.780 × 10^−5^	−0.0991
338.71	20.68	2.205 × 10^−5^	1.990 × 10^−5^	−0.0991
	27.58	3.504 × 10^−5^	3.610 × 10^−5^	0.0316
	34.47	4.395 × 10^−5^	4.990 × 10^−5^	0.1351
	41.37	5.851 × 10^−5^	6.100 × 10^−5^	0.0431
363.15	15.86	1.252 × 10^−5^	9.960 × 10^−6^	−0.2044
	19.53	1.961 × 10^−5^	2.390 × 10^−5^	0.2174
	24.76	4.439 × 10^−5^	5.190 × 10^−5^	0.1684
	27.99	6.528 × 10^−5^	7.080 × 10^−5^	0.0845
	14.14	6.200 × 10^−6^	5.920 × 10^−6^	−0.0455
	18.97	2.400 × 10^−5^	2.130 × 10^−5^	−0.1109
	25.10	6.970 × 10^−5^	5.380 × 10^−5^	−0.2276
	25.10	7.090 × 10^−5^	5.380 × 10^−5^	−0.2407
	32.14	1.017 × 10^−4^	9.490 × 10^−5^	−0.0673
	37.41	1.087 × 10^−4^	1.236 × 10^−4^	0.1374
	40.52	1.260 × 10^−4^	1.392 × 10^−4^	0.1051
366.48	13.79	5.805 × 10^−6^	6.110 × 10^−6^	0.0529
	20.68	3.966 × 10^−5^	3.290 × 10^−5^	−0.1700
	27.58	7.193 × 10^−5^	7.800 × 10^−5^	0.0843
	34.47	1.091 × 10^−4^	1.253 × 10^−4^	0.1487
	41.37	1.530 × 10^−4^	1.685 × 10^−4^	0.1012
383.15	24.83	1.135 × 10^−4^	9.080 × 10^−5^	−0.2001
	32.76	1.894 × 10^−4^	1.921 × 10^−4^	0.0142
	35.41	1.960 × 10^−4^	2.281 × 10^−4^	0.1640
	38.62	2.009 × 10^−4^	2.714 × 10^−4^	0.3509
394.26	20.68	8.716 × 10^−5^	7.570 × 10^−5^	−0.1317
	27.58	1.554 × 10^−4^	1.924 × 10^−4^	0.2382
	34.47	3.153 × 10^−4^	3.489 × 10^−4^	0.1064
	41.37	6.457 × 10^−4^	5.188 × 10^−4^	−0.1966

**Table 7:** Elemental sulfur solubility in CH_4_

*T*/K	*P*/MPa	Solubility (experiment)/mol mol^−1^	Solubility (this model)/mol mol^−1^	Relative error
338.71	27.5792	2.882 × 10^−6^	2.420 × 10^−6^	−0.1588
	34.474	5.357 × 10^−6^	5.450 × 10^−6^	0.0178
	41.3688	8.113 × 10^−6^	1.020 × 10^−5^	0.2511
366.48	20.6844	3.623 × 10^−6^	3.170 × 10^−6^	−0.1241
	27.5792	5.008 × 10^−6^	7.170 × 10^−6^	0.4316
	34.474	1.389 × 10^−5^	1.370 × 10^−5^	−0.0146
	41.3688	2.196 × 10^−5^	2.280 × 10^−5^	0.0400
383.15	20.517	8.100 × 10^−6^	5.750 × 10^−6^	−0.2903
	20.517	6.300 × 10^−6^	5.750 × 10^−6^	−0.0875
	42.414	2.940 × 10^−5^	3.260 × 10^−5^	0.1077
	42.414	2.670 × 10^−5^	3.260 × 10^−5^	0.2197
	50.172	4.080 × 10^−5^	4.780 × 10^−5^	0.1711
394.26	6.8948	2.588 × 10^−6^	2.660 × 10^−6^	0.0282
	20.6844	1.027 × 10^−5^	9.380 × 10^−6^	−0.0861
	27.5792	1.223 × 10^−5^	1.680 × 10^−5^	0.3742
	34.474	2.918 × 10^−5^	2.720 × 10^−5^	−0.0695
	41.3688	4.331 × 10^−5^	4.010 × 10^−5^	−0.0737

## Nomenclature

### Abbreviations

EoS: Equation of state
RE: Relative error
ARE: Average relative error
AARE: Absolute average relative error

### Roman symbols

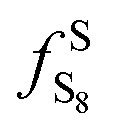

Fugacity of the pure solid specie S_8_ [Pa]

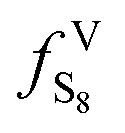

Fugacity of sulfur in vapor phase [Pa]
*P*
: Absolute pressure [Pa]
*T*
: Temperature [K]
*y*
: Sulfur solubility in gas [mol mol^−1^]

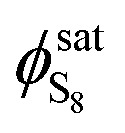

Fugacity factor of sulfur saturation vapor
*R*
: Gas constant [8.314 J (mol^−1^ K^−1^)]

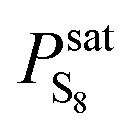

Sulfur saturation vapor pressure at temperature *T* [Pa]

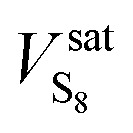

Molar volume of solid sulfur [m^3^ mol^−1^]
*y*_S_8__
: Mole fraction of gaseous sulfur

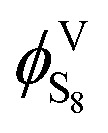

Fugacity coefficient of gaseous sulfur
*V*
: Specific volume [m^3^ mol^−1^]
*T*_c_
: Critical temperature [K]
*P*_c_
: Critical pressure [Pa]
*ω*
: Acentric factor
*T*_r_
: Reduced temperature
*Z*
: Gas mixtures compressibility factor
*n*
: Number of gas component
*y*_*i*_
: Mole fraction in gas mixtures of component *i*
*y*_*j*_
: Mole fraction in gas mixtures of component *j*
*k*_*ij*_
: Binary interaction coefficients between component *i* and component *j*
*Z* ^pred^_*i*_
: Predicting sulfur solubility with model
*Z* ^exp^_*i*_
: Sulfur solubility from experiment
*k*_S_8_–H_2_S_
: Binary interaction coefficient between S_8_ and H_2_S
*k*_S_8_–CO_2__
: Binary interaction coefficient between S_8_ and CO_2_
*k*_S_8_–CH_4__
: Binary interaction coefficient between S_8_ and CH_4_

### Subscripts and superscripts

*i*, *j*: Species index
S: Solid sulfur
V: Vapor
sat: Saturation
r: Reduced state
c: Critical state
pre: Predicting results
exp: Experimental data

## Supplementary Material
